# Nigeria’s 6-year (2018–2023) stage distribution of breast cancer at diagnosis: a systematic review and meta-analysis

**DOI:** 10.3332/ecancer.2025.1899

**Published:** 2025-04-25

**Authors:** Agodirin Olayide, Chijioke Chijindu, Mustapha Fathi, Rahman Ganiyu, Olatoke Samuel, Olaogun Julius, Akande Halima

**Affiliations:** 1 Department of Surgery, University of Ilorin and University of Ilorin Teaching Hospital, Ilorin 240003, Kwara State, Nigeria; 2 Department of Surgery, University of Ilorin Teaching Hospital, Ilorin 240003, Kwara State, Nigeria; 3 Crescent Gold Crown Multispecialist Hospital, Ilorin 240222, Nigeria; 4 Department of Surgery, University of Cape Coast and Cape Coast Teaching Hospital, Cape Coast 000233, Ghana; 5 Department of Surgery, Ekiti State University and Ekiti State University Teaching Hospital, Ado Ekiti 360101, Ekiti State, Nigeria; 6 Department of Radiology, University of Ilorin and University of Ilorin Teaching Hospital, Ilorin 240003, Kwara State, Nigeria

**Keywords:** breast cancer, stage, Nigeria

## Abstract

**Background:**

Nigeria has implemented various interventions to reduce late-stage breast cancer (BC) diagnosis in recent decades. This meta-analysis assessed the impact of these efforts by examining recent BC stage distribution data.

**Methods:**

A systematic review adhering to Preferred Reporting Items for Systematic Reviews and Meta-analysis guidelines was conducted. PubMed was searched for studies on BC in Nigeria from 2018 to 2023 and additional articles were identified through hand searching and snowballing in African Journal Online, Google Scholar and ResearchGate. Data on patient demographics, time to diagnosis at tertiary center and stage distribution were extracted and meta-analyzed using a random-effects model. A simple comparison with historical data of 2000–2018 was conducted.

**Results:**

Eleven articles reported the stage distribution of 1,647 BC patients. Overall analysis of the recent stage distribution showed a slight decrease in stages I, II and IV and an increase in stage III. However, these changes were accompanied by wider confidence intervals: 6% 95% confidence intervals (95% CI 0–15), 17% (6%–29%), 56 (95% CI 38–68) and 21 (9–34) were stages I–IV, respectively, compared to 8% (95% CI 3–13), 21% (14%–28%), 44 (95% CI 33–51) and 29 (21–37) in the historical data. The sensitivity analysis, using a two-stage classification as (‘early’ or ‘late’ disease), strongly indicated a trend towards more advanced-stage (82% CI 79–85) disease in the recent analysis.

**Conclusion:**

Advanced-stage BC remains prevalent in Nigeria. A comprehensive evaluation of current BC control strategies is needed to identify barriers and develop effective interventions for early diagnosis and treatment.

## Introduction/background

Breast cancer (BC) is a significant public health concern. According to the 2024 GLOBOCAN report, the global estimated number of new cases of BC is about 2.3 million, which accounts for 11.6% of cancers in 2022 [1].

WHO data shows significant inequities in the cancer burden, with the effect being more profound in developing countries. Women in high human development index (HDI) countries have a 1 in 12 lifetime risk of BC diagnosis and a BC death rate of 1 in 71 women. In contrast, women in lower HDI countries have a lower lifetime risk of BC diagnosis of 1 in 27 women, yet with a higher expected death rate of 1 in 48 from BC [2].

Nigeria bears a significant burden of BC and has one of the highest age-standardised mortality globally [3]. Over the past years, there has been a substantial increase in the incidence of BC in Nigeria, with the most current available estimate of 54.3 per 100,000 in a 2012 report [4].

A recent meta-analysis involving 23 African countries, encompassing data between 2000 and 2018, shed light on the stage of BC diagnosis across the continent [5], and Nigeria contributed nearly 29% of the included studies. The analysis revealed that advanced-stage tumours were most prevalent in West Africa, with more than 70% advanced-stage disease and Nigeria had the highest proportion of advanced cases, with 91% clinical lymph node positivity [5].

The Nigerian Institute for Cancer Research and Treatment (NICRAT) [6] was established to spearhead cancer control efforts in Nigeria. Their 2018–2022 National Cancer Control Plan aimed to improve access to cancer screening, early detection and timely diagnosis to enhance treatment outcomes. Prior to ‘NICRAT’s establishment and intensifying since then, healthcare providers in the Nigerian healthcare system have implemented various interventions to improve BC awareness, screening programs and access to healthcare services. These initiatives include targeted education campaigns, efforts to improve screening facilities, increased diagnostic tool availability and enhanced healthcare infrastructure [7–12]. These efforts aimed to facilitate early detection and ultimately downstage BC at diagnosis.

Unfortunately, the NICRAT’s executive document outlining the National Strategic Plan for 2023–2027 reported underperformance of the 2018–2022 cancer control plans. In light of these findings, this study aimed to revisit and update the previous meta-analysis by incorporating Nigeria’s most recent and available BC stage distribution data. By doing so, we seek to evaluate the impact of implemented interventions on the stage of BC diagnosis within the country. The current analysis will extend the data for Nigeria from the previous meta-analysis, and a shift towards earlier stages at diagnosis might serve as indirect evidence of the effectiveness of implemented interventions in downstaging BC in Nigeria.

## Methods

This review adhered to the Preferred Reporting Items for Systematic Reviews and Meta-analysis recommendations. After the scoping search, no similar ongoing research was found, so the review was registered on the International Prospective Register for Systematic Reviews (PROSPERO) with ID CRD42023454267.

### Literature search

We conducted full electronic searches in PubMed.gov and supplemented this with hand searching and snowballing techniques. Hand searching involved page-by-page search until page end or saturation in Google, Google Scholar, African Journal Online (AJOL) and ResearchGate to identify additional relevant articles. Snowballing involved reviewing reference lists of articles retrieved after the full electronic and hand search. The search terms ‘BC AND Nigeria’ were applied across all databases and search engines. An initial search opened on 15 August 2023 and closed on 22 August 2023. A supplementary search was conducted between December 21 and 30 December 2023. Where necessary, authors of the included articles were contacted for clarification of missing or obscured data, and the authors’ response window was closed on 5 March 2024, before the final analysis.

### Eligibility criteria

Studies were included based on predetermined PICOTS criteria (detailed in the supplementary PICOTS table). Briefly, eligible studies satisfied the following.

- Population: Reported on female patients with BC in Nigeria.
- Intervention: Not applicable.
- Control: Not applicable.
- Outcomes: Reported the stage of BC at presentation and/or time of arrival at a specialist clinic (primary outcomes).
- Time: Published between 1 January 2018 and 31 December 2023 (articles published in this period but including data before January 1, 2011, were excluded).
- Study design: Original articles with extractable data and a sample size of at least ten participants. There were no language restrictions.

### Study selection

Two independent reviewers (CC and MF) screened the articles using a two-stage process. First, titles and abstracts were screened based on eligibility criteria. Second, full-text articles of potentially relevant studies were retrieved and assessed for final inclusion. Disagreements at any stage were resolved through discussion with a third reviewer (AO).

### Quality of included studies

The quality of the included studies was assessed using a modified version of the Critical Appraisal Skills Programme (CASP) tool [13] (supplementary file) to evaluate five key domains:

1. Validity/Relevance/Applicability: Assessed based on the study’s aim and alignment with the review question.
2. Results presentation and reliability: Assessed based on clarity and consistency of reported findings.
3. Bias: Assessed based on the representativeness of the study population compared to the target population.
4. Precision of staging: Assessed based on the methods used for staging and potential for recall bias.
5. Reporting: Assessed based on the ease of extracting relevant data.

### Statistical analysis

The analysis of the stage at presentation was conducted in two instances. First, a granular analysis focused on individual stages (I–IV). Second, a combined analysis explored the early (I and II) and late (III and IV) stages. To facilitate data aggregation across studies, the proportion of patients in each stage category was calculated by dividing the number of patients in each stage by the sum of patients in all the stages. These proportions were then transformed into summary effect sizes using a random-effects model in the MetaXL add-in for Microsoft Excel (accessible at www.epigear.com). The double arcsine transformation was employed to address potential skewness in proportions. Heterogeneity was assessed using the *I*² statistic, with 75% or higher indicative of substantial heterogeneity.

A similar meta-analytical approach was applied to patient demographics (age, educational status and marital status) and time to a presentation at a specialist clinic, categorised (based on available data) as less than 0–6, 7–12 and >12 months. Age was categorised as <21, 21–30, 31–40, 41–50, 51–60 and >60 years. Educational status was categorised as none, primary, secondary and tertiary, while marital status was categorised as single, married and others (others representing divorced, separated or widowed). Results are presented as percentages with 95% confidence intervals (95% CI). Forest plots for all analyses are included in the supplementary file. Data from articles that did not allow for aggregation were presented narratively.

## Result 

### Article selection

**Figure 1.**
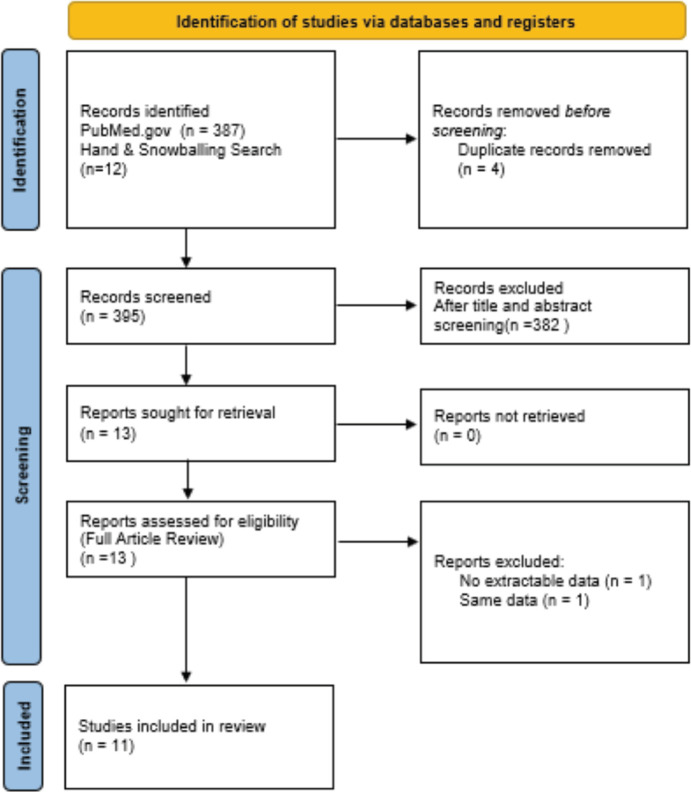
Article selection. Figure 1 shows the article selection process. A total of 399 articles were screened and only 11 were eligible.

The literature search strategy yielded eight articles that met the eligibility criteria. The initial electronic search on PubMed.gov identified 387 articles, of which only three were relevant (14–16). Eight (17–24) additional relevant articles (16–23) were found through supplementary hand and snowballing techniques in Google, Google Scholar, AJOL and ResearchGate. Figure 1 (supplementary file) illustrates the article selection process and Table 1 (supplementary file) summarises the characteristics of the included studies.

### Patient demographics

Six articles [17–19, 21, 22, 24] encompassing data from 751 patients were included in the analysis of age distribution. Three articles (410 subjects) [20, 22, 24], contributed data on educational status and four articles (362 subjects) [19, 20, 23, 24] provided data on marital status.

The modal age group was 41–50 years, representing 31% (95% CI 23–40) of subjects (*I*² = 83%). Nearly half of the subjects had a tertiary education level (47%, 95% CI 21–72, *I*² = 95%) and the vast majority were married (87%, 95% CI 71%–95%, *I*² = 89%). Demographic distributions are shown in Table 2.

### Duration of illness before presentation

Four articles (501 subjects) [17, 18, 22, 24] contributed data to the duration of illness analysis. An estimated 42% (95% CI 22–61, *I*² = 94%) of patients presented within 6 months of detecting the breast change, 37% (95% CI 39–51, *I*² = 94%) presented between 7 and 12 months and 21% (95% CI 15–32, *I*² = 83%) presented after 12 months**.** A single study by Sani [24] provided more granular data for a subset of 100 patients, showing that 3.0% presented within one month and 11% presented between 2 and 3 months.

### Stage of BC at presentation in study (tertiary) centre

**Figure 2.**
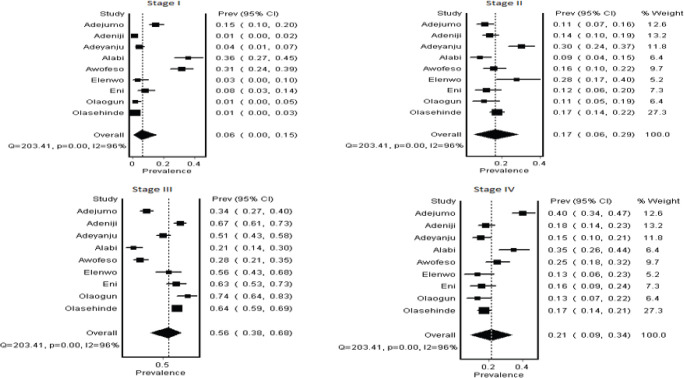
Summary estimate of individual stage of BC recorded at Nigerian study centers (2018–2023 publications).

**Figure 3.**
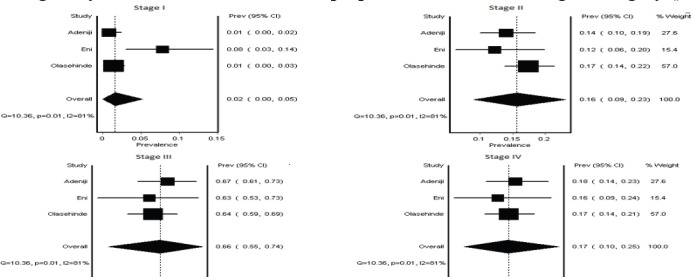
Summary estimate combined stages of BC recorded at Nigerian study centers (2018–2023 publications).

Nine studies (1,482 subjects) [14–22] (contributed to the granular IV stage analysis, while all studies (1,647 subjects) contributed to the two-stage analysis. In the granular individual stages, the most frequent stages at presentation were Stage III (56%, 95% CI 38–68) and Stage IV (21%, 95% CI 9–34), with a high heterogeneity (*I*² = 96%) across studies (Table 3, Figure 2). In the combined Stages**,** when stages were grouped as early (Stage I and II) and advanced (Stage III and IV), 76% (95% CI 67–84) of patients presented with advanced disease (Table 3, Figure 3).

### Sensitivity analysis

To strengthen the reliability of our stage distribution estimates, we conducted a sensitivity analysis focusing on studies with prospective designs and detailed data collection methods description. This analysis included three eligible articles (713 subjects) [16, 18, 22].

**Figure 4.**
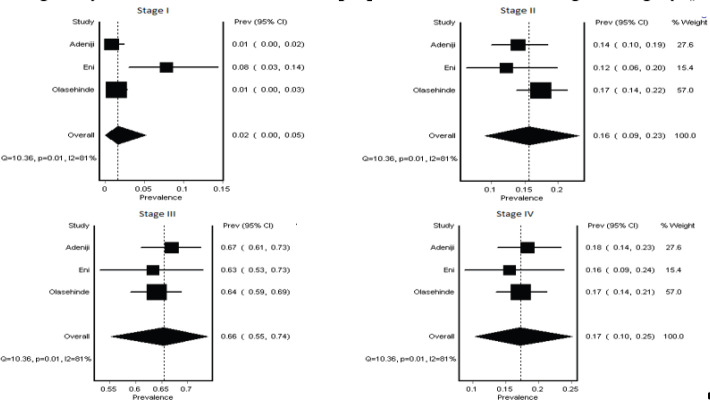
Sensitivity analysis. Figure showing forest plot of the sensitivity analysis of the four-category analysis, shows predominance of late-stage presentation and persistence of high heterogeneity with marked differences in proportions noted in the stage I category.

**Figure 5.**
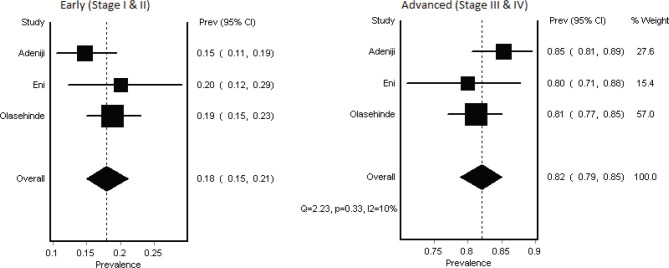
Sensitivity analysis. Figure showing forest plot of the sensitivity analysis of the combined two category staging analysis shows a predominance of late-stage presentation and no heterogeneity.

The results of the sensitivity analysis largely mirrored the overall findings. However, while heterogeneity persisted in the four-category analysis, it was significantly reduced in the two-category analysis. Further examination of the four-category analysis revealed that heterogeneity primarily stemmed from variations in early-stage disease diagnosis. One study reported a notably higher rate of Stage I disease (8%) compared to the other two (1%) (Figures 4 and 5).

## Discussion

This review aimed to describe the summary estimates of the current stage of BC diagnosis in Nigeria, and by comparing current data from 2018 to 2023 with past analyses from 2000 to 2018, we also indirectly evaluated the impact of the current National Strategic Cancer Control Plan (2023–2027) downstaging program.

Despite the similar patient demographics as previously [5], this study’s findings revealed a concerning trend. Aggregate data in the last 6 years showed that the majority of patients were diagnosed after 6 months of symptom detection, and less than 15% presented within 3 months in a more granular single study analysis [24]. In the overall analysis of stage distribution, more than two-thirds (76%, 95%CI 67–84) were diagnosed with advanced stages (III and IV), and only 6% and 17% were diagnosed in stages I and II, respectively, suggesting persistence of challenges in downstaging BC in Nigeria.

To understand how the stage of BC in Nigeria has changed over time, we reviewed available PubMed studies. Data before 2000 was scarce. However, early single studies by Pearson [25] and Chiedozi [26] reported alarmingly high rates of advanced BC, with 95% and 85% of patients, respectively, diagnosed at stages III or IV. More recent aggregate data from 2011 to 2018 showed similar concerning rates (79%) [5]. Our current analysis confirms this trend, with 76% of cases classified as late or advanced in the overall analysis and 82% as advanced or metastatic in the sensitivity analysis. This suggests little improvement in BC stage at diagnosis since the 1980s, aligning with concerns raised by NICRAT about the lack of effectiveness of previous cancer control strategies. Comparing Nigeria to other regions, our estimated metastatic BC rate of 21% falls within the broader range of 5.6% to 30.6% reported for Sub-Saharan Africa (2022) [5, 27].

Our analysis also showed substantial heterogeneity in the data as indicated by high *I*^2^ values, consistent with previous analyses from Africa and Nigeria [5, 28, 29]. In the context of the current study, the high heterogeneity suggests significant variations and probably inaccuracies in diagnostic and staging practices across facilities. This emphasises the need for standardised data collection and reporting practices including developing and implementing a standardised system, training healthcare professionals, and establishing a central cancer registry as part of the NICRAT plan.

Given the reliance on basic imaging modalities such as ultrasound and chest X-ray as the primary staging tools in Nigeria, it is important to optimise the use of these diagnostic tools through the development of context-specific guidelines, training of clinicians and implementing of quality assurance programs to ensure accuracy and reliability of results.

The summary estimate of the BC stage distribution was analyzed using two classification systems: the granular four-stage classification system and the simplified two-stage classification. The summary estimates were more consistent in simplified two-stage classification (early versus late/advanced) than the detailed four-stage system (I–IV). This consistency is likely due to the broader categorisation of the two-stage system, which avoids the complexities and potential subjectivity associated with distinguishing finer gradations within Stages I–IV. Although simplification improves comparability, it sacrifices the detailed and functional information provided by the comprehensive staging system.

The comprehensive 8th Edition of the tumor node metastasis (TNM) staging system, which incorporates biomarkers, offers a detailed cancer profile [30] and aids in treatment selection. However, simplified systems can enhance consistency in diverse settings. Balancing consistency and detail is essential and the optimal approach depends on the context. Simplified systems are valuable for population-level monitoring, whereas the comprehensive TNM system is crucial for individualised treatment planning and prognosis [31], particularly in the early stages. Addressing staging inconsistencies requires the adoption of a standardised staging system, such as a simplified two-stage system for population-level monitoring and the TNM system for individualised treatment as part of the NICRAT plan. Regardless of the system used, diagnostic, staging and reporting criteria should be consistent across the Nigeria.

Inconsistent data collection and reporting practices, as evidenced by wide confidence intervals in this and other studies, hinder effective BC policymaking in Nigeria and Africa. Standardising data collection and reporting will help to address this challenge.

While access to advanced imaging like computed tomography (CT) scans and magnetic resonance imaging (MRI) is limited in resource-constrained settings, healthcare facilities can enhance BC staging by optimally utilising available tools. This includes high-frequency ultrasound, doppler imaging, digital radiography and blood tests, such as liver function tests, serum calcium and phosphate and tumour markers.

Artificial intelligence (AI) offers a promising avenue for improving staging in resource-limited environments [32]. While AI cannot fully replace advanced imaging, it can complement basic diagnostic modalities by providing valuable insights based on available information [32].

With the current trend of advanced computing, machine learning (ML) algorithms and AI can analyze patient data (such as clinical records, laboratory results and basic imaging) and directly map clinical data to stage [32, 33] or indirectly predict cancer stages by mapping clinical data to survival patterns [34]. ML/AI predictions can be preliminary proxies for selecting more expensive high-resolution staging modalities. In the absence of high-end diagnostic modalities, pre-trained deep learning models can be fine-tuned on limited data by using transfer learning [35]. Continuing efforts to expand access to comprehensive diagnostic services will remain essential alongside AI integration.

The new National Strategic Cancer Control Plan (2023–2027) in Nigeria aims to improve BC care by addressing key challenges, including late presentation and stage at diagnosis of BC, limited access to diagnostic tools and inconsistent data collection. For the new NICRAT plan to achieve its aims, a robust monitoring and evaluation framework will be essential to assess progress and make necessary adjustments. Strengthening healthcare infrastructure by improving access to diagnostic equipment, increasing the number of trained personnel and enhancing overall healthcare quality will be crucial for successful implementation.

## Limitations of the study and future directions

High heterogeneity, low-resolution staging modalities and narrow spread of reports are limitations of this review findings. Nonetheless, the findings highlight an urgent need for a comprehensive evaluation to identify key factors hindering progress in downstaging BC in Nigeria. This evaluation should guide the development of new, evidence-based approaches for earlier detection, diagnosis and treatment.

Specifically, research should focus on strategies to increase screening participation and shorten the time between detection, diagnosis and treatment. Strengthening healthcare infrastructure is also essential. This will involve providing necessary diagnostic equipment, well-trained staff and standardised procedures for diagnosis and staging. All strategies should incorporate continuous monitoring and evaluation to ensure ongoing improvements.

## Conclusion

Our analysis of BC data from 2018 to 2023 revealed a persistent pattern of late presentation in tertiary centers and advanced-stage diagnosis in Nigeria. This trend mirrors findings from previous studies spanning two decades. Regarding BC downstaging, the results align with the NICRAT report of 2023, indicating the ineffectiveness of previous strategies to reduce advanced BC cases in Nigeria.

## Conflicts of interest (COI) disclosure

- Agodirin Olayide: No conflicts of interest to declare.
- Chijioke Chijindu: No conflicts of interest to declare.
- Mustapha Fathi: No conflicts of interest to declare.
- Rahman Ganiyu: No conflicts of interest to declare.
- Olatoke Samuel: No conflicts of interest to declare.
- Olaogun Julius: No conflicts of interest to declare.
- Akande Halima: No conflicts of interest to declare.

## Funding/Financial support statement

This research did not receive any specific grant from funding agencies in the public, commercial or not-for-profit sectors.

## Consent for publication

No patient consent was required for this manuscript.

## Ethics approval and consent to participate

No patient consenting required for this manuscript and no approval from any institutional ethics committee required. All included studies are freely available published articles that have satisfied prior ethical considerations and have been duly referenced.

## Availability of data and material

All data used in the analysis are freely available in the studies included. Results of the meta-analysis have been presented in the manuscript or supplementary file.

## Author contributions

All authors contributed to conceiving and developing proposal for this project. AO conducted the article search and registration of proposal, CC, MF and AO contributed to the article selection process. CC and MF extracted data for analysis and AO conducted the statistical analysis. All authors contributed equally to the development and review of the final manuscript.

## Figures and Tables

**Table 1.** Article characteristics.

Author	Study period	Year published	State	Region	Sample size	Design	Modality of staging	Diagnostic criteria
Adejumo *et al* [14]	2015–2018	2019		NC	196	Retrospective	NA	Histo
Adeniji *et al* [22]	2017–2019	2020	Lagos	SW	251	Prospective	AJCC	Histo AJCC
Adeyanju [21]	2016–2019	2020	Lagos	SW	189	Prospective	NA	Histo IHC
Alabi *et al* [19]	2015	2022	Oyo	SW	104	Retrospective	NA	Histo
Awofeso *et al* [20]	2016	2018	Lagos	SW	105	Cross-sectional	AJCC 7	Histo AJCC
Elenwo *et al* [17]	2010–2019	2021	Rivers	SS	61	Retrospective	NA	Histo
Eni *et al* [18]	2014–2016	2019	Ebonyi	SE	89	Prospective	CXR, Bone X-ray, USS positive	Histo
Olaogun *et al* [15]	2010–2015	2020	Ekiti	SW	82	Retrospective	AJCC	Histo
Olasehinde [16]	2015–2018		Osun	SW	372	Prospective		Histo
Sani [24]	2014–2019	2022	Abuja	NC	100	Retrospective	AJCC (NA)	AJCC
Umoke and Garba [23]	2016–2017	2019	Abuja	NC	55	Retrospective		Histo

Table 1 summarises the characteristics of studies included in this review. Most studies were conducted in south west, south south, south east and north-central Nigeria. The study design was mostly retrospective and the modality of staging in most studies was limited to plain chest X-ray and abdominal ultrasound. Abbreviations: AJCC: American Joint Committee on Cancer, CXR: Chest X-ray, Histo: Histopathology, USS: Ultrasound

**Table 2.** Summary estimates of subjects’ demographics.

Demographics						*I* ^2^
**Age distribution (years)**						83%
<21	21–30	31–40	41–50	51–60	>60	
1 (0–2)	6 (2–11)	28 (19–36)	31 (23–40)	20 (13–23)	14 (8–22)	
**Educational status**						
	None	Primary	Secondary	Tertiary		95%
	7 (0–22)	9 (0–26)	37 (13–63)	47 (21–72)		
**Marital status**						
		Single	Married	Others		89%
		6 (0–17)	86 (68–96)	8 (0–22)		

Table 2 shows demographic data. Most subjects fell within the 31–50 age range, and most were married and attained secondary or tertiary education. Abbreviations: Others: Divorced/separated or widow, *I*^2^ = *I*-squared

**Table 3.** Summary estimates of stage at presentation.

Part A	Stage				*I*-squared
Period	I	II	III	IV	*I* ^2^
2018–2023 (All)	6%(0–16)	17% (6–29)	56% (38–68)	21% (9–34)	96%
2018–2023 (Sensitivity)	2%(0–5)	16% (9–23)	66% (55–74)	17% (10–25)	81%
2000–2018 (Historical)	8%(3–13)	21% (14–28)	43% (33–51)	29% (21–37)	93%
**Part B**	Early	Advanced			92%
2018–2023 (All)	24% (16–33)	76% (67–84)			
2018–2023 (Sensitivity)	18% (15–21)	82% (75–85)			10%


Table 3: Comparing summary estimates of stage at presentation for individual stages of BC. Part A shows estimates of four-stage distribution. Part B shows combined stages of presentation as early (stages I and II) and advanced (Stages III and IV). All = all included articles, Sensitivity = included articles eligible for sensitivity analysis only

## Supplementary file

### PICOTS article screening criteria

**Table:**

Participants/Population	Freely available articles found on electronic search or request to corresponding authors, reporting on BC among female Nigeria and including data on stage distribution. Articles reporting on a subpopulation of BC such completed treatment, early, advanced only, premeopausal or premenopausal, recurrence as recurrence, young females, rare cases, metastatic, or early BC alone were excluded.
Intervention	Not applicable
Control	Not applicable
Outcomes	Primary outcome was stage of presentation and time to arrival in specialist clinic. Patient demographics were scondary outcomes Articles not reporting on at least one of the primary outcoems were excluded, secondary data included patient demographics.
Time	We included articles published between January 2018 and December 2023. Articles published in the specified period but including data earlier than January 2011 were excluded.
Study design	The study design was not a strict exclusion criterion provided there was extractable data. The language was also not an exclusion criterion. We included only original articles with an extractable sample size of ten subjects.

### Quality assessment

We modified the CASP tool for quality assessment of the included articles. Five domains were considered as follows

1. Validity/Relevance/applicability: assessed based on the aim
2. Results presentation and reliability: assessed based on the presentation and absecne of descrepancy
3. Bias: assessed based on the popoulation used; all or purposeive or subset
4. Precision of staging : asseses based on the modality of staging and attempt to prevent recall bias
5. Reporting: Assessed based on ease of extraction.

**Table:**

Validity/Relevance/APPlicability: assessed based on aim and rational	Aimed to describe stage/pattern of presentiaotn 2.0	Other aim/rationale 1.0	Not reported 0
design	Pros-2	retro 1.0	Not stated 0
Bias	All population :2		Subpopulation 1.0
Precision	CT included : 2.0	Stated Cxr, USS, FBC, LFT 1.0	Clinical or not stated 0.5
Reporting	No recalc 2.0		Recalc = 1.0

**Table:**

Author	Rational staging (max2)	Design proposed (max 2)	Population (max 2)	Staging (max 2)	Reporting (max 2	Qualityscore (max 10)
Adejumo	2	1	2	0.5	1	6.5
Adeniji	1	2	Acutely Ill Excluded (1)	0.5	1	5.5
Adeyanju	2	2	Acutely Ill Excluded (1)	0.5	1	6.5
Alabi	1	1	2	0.5	1	5.5
Awofeso	2	2	1	0.5	1	6.5
Elenwo	2	1	2	0.5	1	6.5
Eni	1	2	2	1	1	7
Olaogun	2	1	2	1	1	7
Olasehinde	2		2	1	1	8
Sani	2	1	2	0.5	1	6.5
Umoke	1	1	2	0.5	1	5.5
